# The Effects of Cross-Legged Sitting on the Trunk and Pelvic Angles and Gluteal Pressure in People with and without Low Back Pain

**DOI:** 10.3390/ijerph17134621

**Published:** 2020-06-27

**Authors:** Kyoung-sim Jung, Jin-hwa Jung, Tae-sung In

**Affiliations:** 1 Physical Therapy, Gimcheon University, Gimcheon 39528, Korea; jkspt@hanmail.net; 2 Occupational Therapy, Semyung University, Jecheon 390-711, Korea; otsalt@nate.com

**Keywords:** cross-legged sitting, trunk flexion angle, pelvic obliquity, gluteal pressure

## Abstract

The purpose of this study was to investigate the effects of cross-legged sitting on the trunk flexion angle, pelvic obliquity, and gluteal pressure of subjects with and without low back pain (LBP). The study subjects were 30 LBP patients and 30 healthy individuals. They were instructed to sit on a chair, the height of which was adjustable, so that their knee and hip joints were bent at 90°. All subjects were asked to perform two sitting postures: erect sitting and cross-legged sitting. Trunk flexion angle and pelvic obliquity were measured using a three-dimensional motion-capture system, and gluteal pressure was measured using a force plate. Compared to erect sitting, cross-legged sitting showed a significantly lower trunk flexion angle and greater pelvic obliquity in both groups. Compared to healthy subjects, the patients with LBP had lower trunk flexion angles and greater gluteal pressure asymmetry during cross-legged sitting. The pelvic obliquity was greater in the cross-legged sitting posture than in the erect sitting posture, but there was no difference between the groups. We found that the trunk became more slouched in the cross-legged sitting posture than in the erect sitting posture, and this tendency was more pronounced in patients with LBP.

## 1. Introduction

Adolescents and adults spend an average of 7.7 h a day sitting [1]. The lordosis in the sitting posture decreases compared to that in the standing posture [2,3]. Sitting in an upright posture for a prolonged period without support can be difficult, as well-balanced trunk muscle strength and endurance are required to maintain proper posture [4]. Sitting for an extended period in an uncomfortable position can possibly lead to an increase in joint load, causing various musculoskeletal diseases, including pain [5,6].

Studies that investigated the natural sitting posture of patients with low back pain (LBP) showed decreased lumbar lordosis and increased cervical lordosis and thoracic kyphosis compared to erect sitting postures [7]. Studies comparing the sitting postures of subjects with and without chronic LBP reported that the LBP group showed an asymmetrical distribution of body weight [8] and decreased activity of the internal obliques [9] compared to the control group. Fann [10] compared posture asymmetry in patients with LBP in a standing posture and observed no significant difference in pelvic obliquity between subjects with and without chronic lower back pain.

Many individuals often sit with one leg crossed during their daily lives. Cross-legged sitting provides the physiological benefits of reducing muscle fatigue by decreasing the activity of the external and internal obliques [11,12], and it contributes to joint stability by compressing the sacroiliac joints [12]. However, hip flexion and adduction are required to maintain the cross-legged sitting posture. As a result, the rotation of the spinal column is increased due to the pelvic rotation, and muscle length and strength are changed, which may cause musculoskeletal pain [13]. Lee et al. [14] reported that the craniocervical angle increased and the trunk flexion angle decreased during continuous cross-legged sitting. Furthermore, Yu et al. [15] compared the pelvic angle in different sitting postures and showed that pelvic obliquity and posterior tilt angle were significantly increased in the cross-legged sitting posture compared to the erect sitting posture.

However, most of the studies that have examined sitting postures were conducted on healthy subjects, and studies that have elucidated the sitting postures of patients with LBP are insufficient. In addition, the differences in the trunk and pelvic angles during cross-legged sitting, which increases pelvic obliquity, between subjects with and without chronic LBP have not yet been investigated.

Therefore, the current study aims to compare differences in the trunk flexion, pelvic obliquity, and gluteal pressure during cross-legged sitting between subjects with and without nonspecific LBP.

## 2. Subjects and Methods

### 2.1. Participants

Thirty patients (22 males and 8 females) with LBP and 30 controls (20 males and 10 females), aged between 22 and 34 years, were included in the study. We recruited patients who presented with a first episode of mechanical LBP of more than 3 months’ duration. The control group included individuals with no previous history of LBP. The exclusion criteria were anamnesis of medical or drug abuse, surgery on the musculoskeletal system, history of neurological disorder, tumor, infection, or inflammatory arthropathy. Informed consent was voluntarily obtained from all subjects before participation in our study, which was approved by the Institutional Review Board (IRB) of Gachon University (IRB No. 1044396-201801-H13-009-01).

### 2.2. Protocol

In this study, subjects were instructed to sit without support on a height-adjustable table with a force plate at the top and maintain the posture for one minute. The order of upright sitting and cross-legged sitting was randomly presented. When adopting a cross-legged sitting posture, the dominant knee was crossed over the other knee. The predominant leg was determined to be the one used to kick a ball. Before performing the measurements, the height of the chair for each patient was adjusted to ensure their knee flexion angle was 90°. Trials were repeated three times, for one minute per posture. Five-minute rests were granted between trials to reduce fatigue problems. In order to acquire data while the posture was stably maintained, data collected during the first and last 10 s were excluded from the analysis.

### 2.3. Outcome Measurements

**Figure 1:**
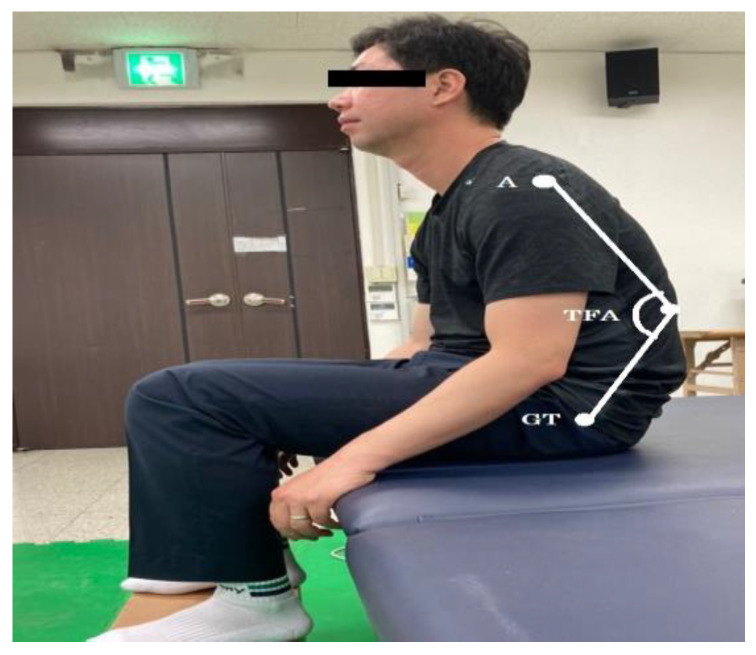
Measurement of trunk flexion angle. A: acromion, TFA: trunk flexion angle, GT: greater trochanter.

Reflective markers were attached to the acromion, spinous process of the first lumbar vertebra (L1), mid-point of the greater trochanter, and both anterior superior iliac spines (ASISs). Trunk flexion angle and pelvic obliquity were measured and recorded using a motion capture system with ten infrared cameras (Raptor-E, Motion Analysis Inc., Santa Rosa, CA, USA), at a sampling rate of 100 Hz. Kinematic data were analyzed using video-motion analysis software named ORTHOTRAK (6.2.4, Motion Analysis Inc., CA, USA). The trunk flexion angle was measured based on the angle between the line connecting the left acromion and L1 spinous process and the line connecting the L1 spinous process and left greater trochanter [14,16] (Figure 1). 

Frontal plane asymmetry, commonly known as pelvic obliquity, in which one innominate bone is higher or lower than the other innominate [17], was calculated according to the angle between the horizontal plane defined by the global coordinate system of the motion capture volume and the line connecting both ASISs [18]. 

To acquire the gluteal pressure data, a force plate (9286B, Kistler, Winterthur, Switzerland) was used. This equipment comprises piezoelectric 3-component force sensors that enable researchers to obtain an accurate center of pressure and low crosstalk values. The sample frequency was set to 1200 Hz. Peak pressure means the greatest pressure values from the distribution around the ischial tuberosity. The force plate was divided into two regions (left and right) and the pressure distribution for each region was analyzed using the MatLab™6 software (The MathWorks, Inc., Natick, MA, USA). The peak pressure ratio was calculated as the ratio of the higher peak pressure side to lower peak pressure side. A higher value indicates a more asymmetric sitting posture [8]. The mean trunk and pelvic angles and maximum gluteal pressure for the middle 40 s were analyzed. The average value of three measurements and kinematics were used for the analysis. The Numeric Pain Rating Scale (NPRS) was used to measure pain intensity in LBP patients. The NPRS is a measure that can express the level of pain that one feels in ten steps, which means that the higher the score, the more severe the pain [19]. Patients with LBP were instructed to rate the level of pain they felt during daily life.

### 2.4. Data Analysis

SPSS 21.0 was used for statistical analysis. The normality of variables was assessed using the Shapiro–Wilk test. The independent *t*-test for continuous variables (age, height, and weight) and the chi-square test for categorical variables (e.g., sex) were used to compare the general characteristics of the subjects in the LBP and control groups. The effects of sitting posture, group, and their interaction on trunk flexion angle, pelvic obliquity, and peak pressure ratio were examined using a two-way analysis of variance (ANOVA) for repeated measures. When a significant interaction between independent variables was detected, the effect of each variable was examined separately using a paired *t*-test (for investigation of the effect of sitting posture in each group) and an independent *t*-test (for the investigation of the effect of group on each sitting posture). The level of statistical significance was set at 0.05.

## 3. Results

### 3.1. General Characteristics of Subjects

Table 1 shows the characteristics of the participants in each group. There was no significant difference in any of the characteristics of the participants.

### 3.2. Comparison of Trunk Flexion Angle

**Figure 2:**
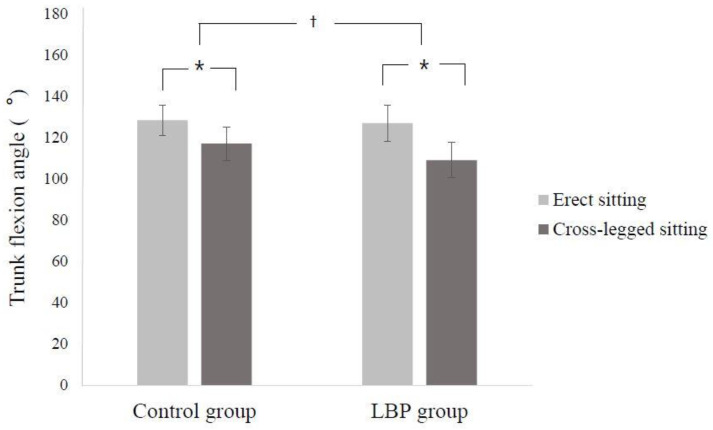
Mean (standard deviation) of trunk flexion angle during two different sitting postures. * Significantly different within the group. ^†^ Significantly different between groups.

There were significant differences between the two groups in the change in trunk flexion angle according to posture (interaction effect between group and sitting posture: F = 16.959, *p* = 0.000) (Figure 2). 

Thus, follow-up analyses were performed using *t*-tests to investigate the effect of sitting posture within each group and the effect of the group for each sitting posture.

Simple main effect analyses of the trunk flexion angle revealed that the trunk flexion angle of cross-legged sitting was significantly decreased in both groups compared to erect sitting (t = 12.895, *p* = 0.000 for LBP group; t = 13.413, *p* = 0.000 for control group). Moreover, the trunk flexion angle was not significantly different between the groups when sitting in an upright position (t = 0.644, *p* = 0.522), but the trunk flexion angle of the LBP group was significantly decreased compared to the control group when sitting with legs crossed (t = 3.458, *p* = 0.001).

### 3.3. Comparison of Pelvic Obliquity

**Figure 3:**
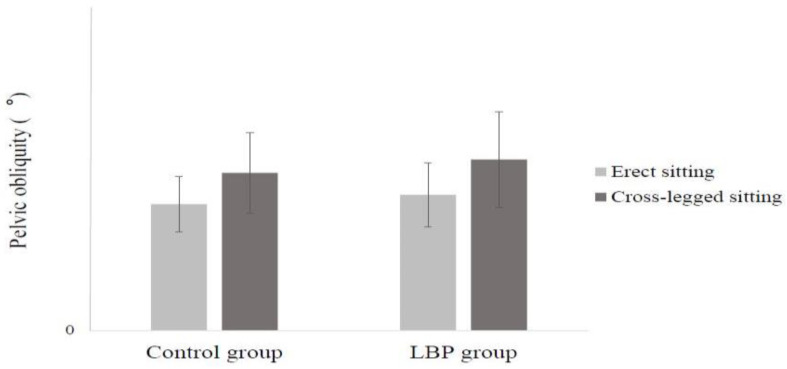
Mean (standard deviation) of pelvic obliquity during two different sitting postures.

It was found that the pelvic obliquity of all participants was significantly greater in the cross-legged sitting posture than in the erect sitting posture (F = 29.118, *p* = 0.000), but there was no significant difference between the groups (F = 2.184, *p* = 0.145). There were no significant differences between groups in the change in pelvic obliquity according to posture (interaction effect between group and sitting posture: F = 2.184, *p* = 0.145) (Figure 3). 

### 3.4. Comparison of Peak Pressure Ratio

**Figure 4:**
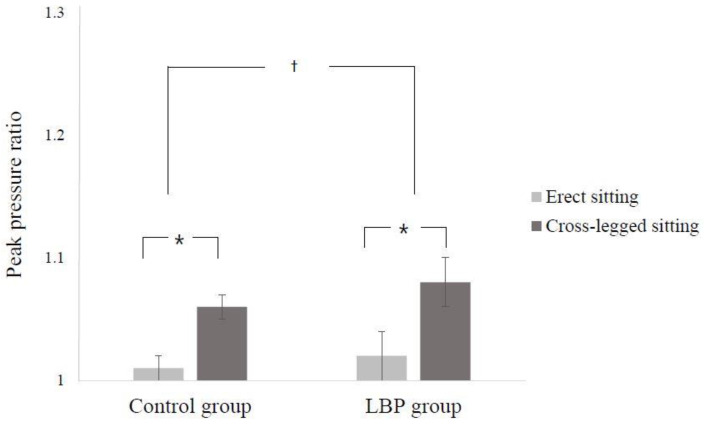
Mean (standard deviation) of peak pressure ratio during two different sitting postures. * Significantly different within the group. ^†^ Significantly different between groups.

There were significant differences between groups in the change in peak pressure ratio according to posture (interaction effect between group and sitting posture: F = 6.938, *p* = 0.011) (Figure 4). 

Thus, follow-up analyses were performed using *t*-tests to investigate the effects of sitting posture within each group and the effects of the group for each sitting posture.

Simple main effect analyses of the peak pressure ratio revealed that the peak pressure ratio of cross-legged sitting was significantly decreased in both groups compared to the erect sitting posture (t = −16.268, *p* = 0.000 for LBP group; t = −16.378, *p* = 0.000 for control group). Furthermore, the peak pressure ratio was not significantly different between the groups when sitting in an upright position (t = −1.231 *p* = 0.223), but the peak pressure ratio of the LBP group was significantly decreased compared to the control group when sitting with legs crossed (t = −3.622, *p* = 0.001).

## 4. Discussion

The current study compared the differences in trunk flexion angle in two different sitting postures in subjects with and without nonspecific chronic LBP. The results indicated that there was a significant difference between the two groups in the change in trunk flexion angle according to posture. In cross-legged sitting, the trunk flexion angle of the LBP group was significantly reduced compared to the control group, which means that the posture of the LBP group during cross-legged sitting was more slumped. Keegan [20] reported that when sitting for a long time, the most important factor in the development of LBP is a decrease in the lordosis of the lumbar spine. Murphy et al. [21] reported that a flexed posture is significantly correlated with LBP. Studies on sitting posture in patients with LBP also showed that cervical lordosis and thoracic kyphosis increased while sitting naturally compared to erect sitting [7]. One study divided the LBP group into two subgroups according to the posture of worsening pain, and then compared the difference in natural sitting posture, between these subgroups and healthy subjects. According to the results, compared to healthy adults, there was a decrease in lumbar lordosis in the group in which the pain worsened in the lumbar flexion posture, and the opposite result was observed in the group in which the pain was exacerbated in the lumbar extension posture. They also suggested that the subjects had this posture before the onset of LBP; hence, it was due to a decrease in postural control ability rather than a reflexive response to pain [22]. Patients with LBP tend to have reduced proprioception of the lumbar spine [23], and the ability to maintain equilibrium around the “neutral zone” decreases [24]. Therefore, while sitting, the lumbar spine is positioned away from the neutral zone, resulting in increased tissue deformation and tissue damage [25]. In addition, Dankaerts et al. [26] compared the activity of trunk muscles in the sitting positions of LBP patients and healthy subjects. They found that LBP patients whose symptoms were exacerbated during lumbar flexion had decreased activation of local stabilizing muscles compared to healthy adults, and LBP patients with exacerbation of symptoms during lumbar extension had increased co-activation of these muscles. They also reported that this change in muscle activity caused pain and a maladaptive postural pattern. More than 90% of the patients with LBP who participated in this study indicated that the symptoms worsened during lumbar flexion. Accordingly, as the trunk flexion angle of the LBP patients in the cross-legged sitting posture significantly decreased compared to the control group, this finding was consistent with that of Dankaerts et al., who observed a kyphotic sitting posture in LBP patients whose symptoms exacerbated during lumbar flexion compared to healthy adults [22]. These results suggest that the activity of the trunk muscles is reduced in cross-legged sitting [11,12], so that kyphotic posture is more pronounced in LBP patients who have decreased trunk control. However, the erect sitting posture includes less lumbar lordosis and a relaxed thorax, and it is thought that the differences between the groups may have decreased as a result of subjects trying to sit more upright than usual.

In addition, this study analyzed the effects of cross-legged sitting posture on pelvic obliquity. In both groups, cross-legged sitting led to a significant increase in pelvic obliquity compared to erect sitting. However, there was no difference between the two groups in the change in pelvic obliquity according to posture. In a study comparing pelvic obliquity and gluteal pressure according to sitting posture [15], pelvic obliquity was increased when the legs were twisted. In addition, gluteal pressure increased as the weight of the upper body was transferred to the uncrossed leg’s side. However, studies on the increase in pelvic asymmetry in patients with LBP were mainly performed on the sagittal plane [27]; there were no significant differences in studies comparing pelvic obliquity between back pain patients and healthy adults [10]. In this study, pelvic obliquity was compared in cross-legged sitting, where postural asymmetry increased, but there was no significant difference between groups, as in the previous study. However, the peak pressure ratio, which is the index of asymmetrical sitting posture, was significantly higher in the LBP group than in the control group, during cross-legged sitting. The weight of the upper body is mainly transferred to the ischial tuberosity [28], and gluteal pressure is influenced by the sitting posture [29,30]. In this study, the significant increase in the peak pressure ratio of LBP patients in cross-legged sitting was thought to be more difficult to control than the posture of LBP patients, because cross-legged sitting increases pelvic asymmetry as well as pelvic obliquity compared to erect sitting. Schamberger [13] reported that crossing the legs causes pelvic rotation, which in turn increases rotation in the lumbar spine. Although pelvic obliquity did not differ significantly between groups in this study, it is thought that pelvic asymmetry increased as a result of combining other factors, such as pelvic and lumbar rotation, which were not measured in this study. In this study, there was no significant difference in peak pressure ratio between the two groups in erect sitting. This is because, unlike previous studies that measured it in a natural sitting posture, peak pressure ratio was measured in the erect sitting posture; another reason is that the measurement time was short. Patients with LBP tend to have reduced trunk motor variability during low intensity activities [31,32], and this tends to fatigue the back muscles easily [33]. Furthermore, due to the ligamento-muscular protective reflex [34,35], the flexion relaxation ratio was significantly decreased compared to healthy adults [26]. Therefore, prolonged sitting in an asymmetrical posture increases back pain and lumbar discomfort [22,36,37,38]; thus, it can have a more detrimental effect on posture control.

This study compared the differences in trunk and pelvic angles and gluteal pressure according to sitting posture in patients with or without back pain. As a result, it was observed that the trunk flexion angle was significantly decreased, and the gluteal pressure ratio was significantly increased, in patients with LBP compared to the control group. This is consistent with the results of previous studies, in which the postures of LBP patients were more slumped and asymmetrical. However, this study was limited since the number of subjects was small. Furthermore, the subjects were sitting on a rigid force plate without a backrest during the measurements in this study; this may have influenced their sitting postures. Therefore, there are several limitations to generalizing the results of this study. In addition, other pelvic asymmetry factors, including pelvic rotation, were not evaluated in this study. Future studies need to increase the number of subjects and analyze the differences in muscle fatigue and various trunk and pelvic angle changes after prolonged sitting between patients with various patterns of LBP and healthy adults.

## 5. Conclusions

In conclusion, the results of this study suggest that cross-legged sitting leads to a bent and asymmetrical posture, and this effect is more pronounced in patients with LBP.

## Figures and Tables

**Table 1:** Common and clinical characteristics of the subjects (*N* = 60).

Variables	LBP Group (*n* = 30)	Control Group (*n* = 30)	*p*
Sex (Male/Female)	22/8	20/10	0.779 ^b^
Age (years)	24.43 ± 2.73 ^a^	24.17 ± 2.77	0.708 ^c^
Height (cm)	170.47 ± 7.53	171.37 ± 9.19	0.684 ^c^
Weight (kg)	65.50 ± 12.79	66.90 ± 10.80	0.649 ^c^
NPRS	4.90 ± 0.96	0.0 ± 0.0	
Postures that make symptoms worse (lumbar flexion/extension)	(27/3)		

^a^ Mean ± standard deviation, ^b^ chi-square test, ^c^ independent *t*-test. LBP; Low back pain, NPRS; numeric pain rating scale.
